# Impact of COVID-19-Mediated Olfactory Loss on Quality of Life

**DOI:** 10.1159/000523893

**Published:** 2022-04-12

**Authors:** Martin Sylvester Otte, Antje Haehner, Marie-Luise Bork, Jens Peter Klussmann, Jan Christoffer Luers, Thomas Hummel

**Affiliations:** ^a^Department of Otorhinolaryngology, Head and Neck Surgery, Faculty of Medicine and University Hospital Cologne, University of Cologne, Cologne, Germany; ^b^Department of Otorhinolaryngology, Smell and Taste Clinic, Technische Universität Dresden, Dresden, Germany; ^c^Center for Molecular Medicine Cologne (CMMC), University of Cologne, Faculty of Medicine and University Hospital Cologne, Cologne, Germany

**Keywords:** Coronavirus disease 2019, Severe acute respiratory syndrome coronavirus 2, Smell disorders, Quality of life, Sniffin' sticks

## Abstract

**Introduction:**

COVID-19 can be associated with a variety of longer-lasting impairments that can have a significant impact on patients' quality of life (QoL). While this is well described in the literature for limitations in lung capacity or permanent headaches, there is little research on the impact of olfactory dysfunction in the context of COVID-19 on patients' QoL.

**Methods:**

In 65 patients with a history of COVID-19, the present olfactory ability was assessed using the Sniffin' Sticks test. In addition, olfactory QoL was assessed by the Questionnaire of Olfactory Disorders. Self-assessment was performed with visual analogue scales. The data were compared with the results obtained in healthy individuals and in patients with hyposmia due to other viral infections.

**Results:**

The QoL of COVID-19 patients was significantly lower compared to the healthy control group. Even recovered subjects whose olfaction had already returned to the normal range still had a reduced QoL. The severity of the olfactory impairment correlated with the reduction in QoL. However, the olfactory QoL of COVID-19 patients was not worse than that of patients' olfactory loss due to other viral infections. Patients with parosmia had reduced QoL and rated their situation worse than patients without parosmia.

**Conclusion:**

QoL appears to be impaired in patients with long-lasting COVID-19 olfactory disorders several months after overcoming acute symptoms, even if olfaction has normalized. However, the impairment is not more pronounced than in patients with other postviral olfactory disorders of the same duration.

## Introduction

Olfactory dysfunction is a common symptom of COVID-19 [1, 2, 3]. While the impairment lasts only a few weeks in the majority of most affected patients [4], it may persist for several months in others and even after half a year, a low olfactory sensitivity is found in some of those who have recovered from the disease [5, 6]. Therefore, olfactory dysfunctions is also listed as part of the secondary disease “long-COVID” after an infection with SARS coronavirus-2 [7].

Acquired olfactory disorders can be associated with a subjective reduction in quality of life (QoL), as recently confirmed in a large multicenter study [8]. The cause of the olfactory disorder, for example chronic sinusitis, infection, or trauma, has a specific impact on QoL [8]. The aim of the present study was to investigate to what extent the COVID-19-related loss of smell affects the patients' QoL and whether this possible impairment is more severe than in olfactory disorders due to other viral infections of the upper respiratory tract.

## Methods

This study was conducted at a university hospital in Germany. The principles of the World Medical Association Declaration of Helsinki and its subsequent amendments were followed and adherence was monitored by the Local Ethics Committee. Patients were recruited from the olfactory outpatient clinic for COVID-19 recovered patients and gave their written informed consent to participate in the study. The disease was initially confirmed by polymerase chain reaction and there was no need for hospitalization of any of the participants during the course of the disease. All patients had been recovering from the acute disease for at least 2 months and had reached the age of 18. Preexisting nasal diseases such as chronic sinusitis, acute allergies, trauma to the nose, or known olfactory and gustatory disorders prior to the disease were denied by all subjects. Structured questionnaires were used to collect this information, as well as other sociodemographic data such as age and gender.

The data of the control groups were taken from an existing database. The controls were 32 healthy volunteers with normal olfactory function and 14 hyposmic patients who had previously been treated for postviral (not COVID associated) olfactory disorders. Orthonasal olfactory ability of the patients was measured using the felt-tip pen-based Sniffin' Sticks Test (Burghart Messtechnik GmbH, Pinneberg, Germany). The test allows to determine the olfactory threshold, the ability to distinguish odors, and the ability to identify odors separately. The results are presented as a composite TDI score, where values below 16.5 points correspond to functional anosmia, values between 16.5 and 30.5 points to hyposmia, and values above 30.5 points to normosmia [9].

The subjects' QoL was assessed using the German version of the Questionnaire of Olfactory Disorders (QOD), a widely used and well-validated test of olfactory-related QoL [10]. This test consists of various statements that the participants agree with, partially agree with, partially disagree with or cannot agree with. Depending on the question and the patient's answer, the participant's statement was scored from 0 to 3 points. Nineteen statements refer to QoL (QOD-QOL), 4 to possible parosmia (QOD-P) and 6 questions refer to social desirability (QOD-DS). Depending on the answer, scores of 0–3 are given, so that 0–57 are scored for the QOD-QOL part and 0–12 for the QOD-P part. A higher score indicates a more severe impairment on these parts of the QOD. QOD-DS scores ranged from 0 to 18. A higher score indicates that the participant is more likely to give a socially desirable − and thus less reliable − answer. In addition, the subjective limitation of the patients by their olfactory disorder was evaluated by 5 visual analogue scales (QOD-VAS), on which the subjects are asked to classify their problems at work, in family and social life with regard to their olfactory impairment. The range of the scales was defined from “not at all” (0 units) on the left to “always” (10 units) on the right, so that a higher value indicates a stronger impairment.

Statistical analysis was performed using SPSS v. 24 (SPSS Inc., Chicago, IL, USA). Data are presented as means (± standard errors of the means). The mean values were compared after Levene's test for equality of variance via a *T* test for independent samples. The significance of the differences between the genders of the respective groups was determined with the χ^2^ test. The correlations were calculated according to Spearman-Rho, the two-sided significance of the coefficients of correlation is presented.

## Results

Sixty-five post-COVID patients were included in the study with a mean age of 44.9 years (±11.7 years). The group consisted of significantly less men (*n* = 24, 37%) than women (*n* = 41, 63%) (*p* = 0.035). The healthy control group (healthy controls) consisted of 19 women and 13 men (*n* = 32 in total, gender balanced, *p* = 0.29) with an average age of 51.8 years (±15.0 years). The control group of patients with hyposmia due to other viral non-COVID-associated infections of the upper respiratory tract (postviral controls) consisted of 2 men and 12 women (*n* = 14 in total, not balanced for gender, *p* = 0.008) with an average age of 58.6 years (±11.32 years).

There was no significant difference in gender distribution between the post-COVID patients and the healthy controls (*p* = 0.78) and between the post-COVID patients and the postviral controls (*p* = 0.59). There was also no significant difference in gender distribution between the control groups (*p* = 0.053). In age, the post-COVID patients differed significantly from the healthy controls (*p* = 0.015) and from the postviral controls (*p* = 0.001). There was no significant age difference between the control groups (*p* = 0.13).

Since the acute infection, 7.1 months had passed in the COVID patients and 6.4 months in the postviral controls. This difference was not significant (*p* = 0.30).

### Olfactory Function − Psychophysical Measures

The mean TDI of the post-COVID patients was 32.9 (±0.9). Fifty-three (82%) of the participants had TDI scores in the normosmic range and 10 (15%) were hyposmic. Because only 2 (3%) of the participants scored in the functionally anosmic range, these were included in the hyposmic group for calculations (hereafter referred to collectively as “hyposmic post-COVID patients” for simplicity). This was also based on the idea that there is a continuum in olfactory function between hyposmia and functional anosmia. The mean TDI within this group was 21.98 (±2.77) (Table 1).

**Fig. 1:**
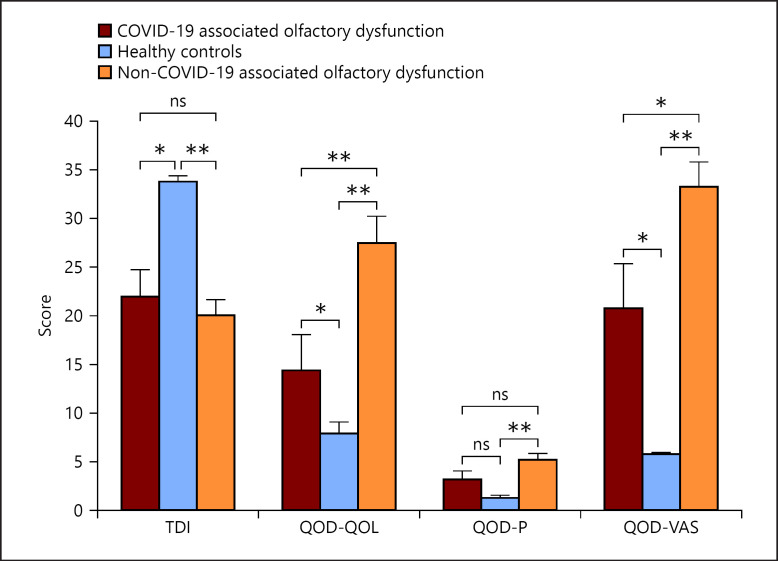
Comparison between patients with COVID-19-associated olfactory dysfunction, healthy controls, and patients with non-COVID-19-associated olfactory dysfunction due to other viral infections regarding olfactory function (TDI-Score) and the subitems of the QOD. Items regarding QOD-QOL, QOD-P, and QOD-VAS were asked. ns, not significant, *: *p* < 0.05, **: *p* < 0.01.

In the healthy controls, the TDI was in the normosmic range for all individuals and averaged 33.8 (±0.53) (Table 1). The difference to the post-COVID patients was not significant overall (*p* = 0.49), but to the hyposmic post-COVID patients (*p* = 0.001) (Fig. 1). In the postviral controls, a mean TDI of 20.04 (±1.59) was obtained (Table 1) which was not significantly different from the hyposmic post-COVID patients (*p* = 0.53) (Fig. 1).

### QoL Measurements − QOD Questionnaire

Compared to the healthy controls, post-COVID patients had slightly higher QOD-QOL scores, although this effect was not significant. However, within the COVID-19 group, hyposmic post-COVID patients had a score of 14.38 (±3.70), almost double than that of the normosmic post-COVID patients (7.69, *p* = 0.059) and healthy controls (7.94, *p* = 0.034) (Table 1). The difference between the hyposmic post-COVID patients and the healthy controls was significant (*p* = 0.034) (Fig. 1). The postviral controls had a QOD-QOL score of 27.43 (±2.79) (Table 1) which was significantly higher compared to the hyposmic post-COVID patients (*p* = 0.011) (Fig. 1).

### QoL Measurements − Parosmia

The score for QOD-P was 2.50 (±0.41) for all post-COVID patients. Within the post-COVID patients, the normosmics had a score of 2.36 (±0.46) and the hyposmics had a score of 3.13 (±0.93) (Table 1). The healthy controls had a mean QOD-P value of 1.28 (±0.33), the postviral controls a value of 5.07 (±0.75) (Table 1). Thus, while there was a significant difference between the post-COVID patients overall and the healthy controls (*p* = 0.024), there was no such difference (*p* = 0.096) between the hyposmic post-COVID patients and the healthy controls (Fig. 1). Also, the difference in QOD-P between the hyposmic post-COVID patients and the postviral controls was not significant (*p* = 0.127) (Fig. 1).

### QoL Measurements − VAS Ratings

In the QOD-VAS, post-COVID patients scored 11.34 points (±1.60), with normosmic post-COVID patients scoring 9.25 (±1.49) and hyposmic post-COVID patients scoring 20.75 (±4.57) (Table 1). The difference between the subgroups of the post-COVID patients was highly significant (*p* = 0.001) (Fig. 1). The healthy controls had scores of 5.78 (±0.16) points, the postviral controls 33.25 (±2.56) (Table 1). Thus, both the normosmic post-CO­VID (*p* = 0.027) and the hyposmic post-COVID patients (*p* = 0.014) had significantly higher scores than the healthy controls. There was also no significant difference in QOD-P between the hyposmic post-COVID patients and the postviral controls (*p* = 0.13) (Fig. 1).

### QoL Measurements − QOD-DS

In the QOD-DS, post-COVID patients scored 8.48 points (±0.33), with normosmic post-COVID patients scoring 8.47 (±0.39) and hyposmic post-COVID patients scoring 8.5 (±0.46) (Table 1). The difference between the subgroups of the post-COVID patients was not significant (*p* = 0.96) (Fig. 1). The healthy controls had scores of 11.69 (±0.55) points, the postviral controls 11.25 (±1.04) (Table 1). The differences between the post-COVID patients and the healthy controls (*p* = 0.001) as well as between the post-COVID patients and the postviral controls (*p* = 0.001) were significant, whereas there was no significant difference between healthy controls and postviral controls (*p* = 0.69).

### Correlations

Within the group of post-COVID patients, there was a negative correlation between the TDI score and the scores in the QOD-QOL (*r* = −0.35, *p* = 0.020) as well as in the self-assessment visual analogue scale (*r* = −0.31, *p* = 0.038). QOD-QOL (*r* = 0.57, *p* < 0.001) and QOD-VAS (*r* = 0.59, *p* < 0.001) also correlated with the scores in the QOD-P.

## Discussion

COVID-19 has a number of long-term sequelae that extend beyond the acute infection and, as far as it can be predicted so far, last for months and in a certain percentage of patients, even longer. COVID-associated olfactory loss remains detectable for more than 6 months in a large number of patients [6] and even in the long-term course, limitations in the ability to smell seem to persist in about 7% of those affected [11]. When assessing olfactory function with validated psychophysical tools more than 1 year after the infection, an olfactory impairment seems to be present in more than 40% of these patients [12]. Given the high number of people suffering from COVID-19, a substantial number of people worldwide will therefore be left with a severe olfactory dysfunction.

Patients with acquired olfactory disorders often show an impaired QoL [8] and it is known that this olfactory QoL correlates with the general QoL [13]. Compared to other olfactory disorders, such as sinunasal olfactory disorders, postinfectious olfactory dysfunction is associated with a higher level of subjective impairment [8, 14]. In the present study, we showed that individuals recovering from COVID-19 still experience limitations in their QoL even several months after the acute phase of the disease. Within post-COVID patients we found an association between impairment of olfactory function and QoL − the lower the TDI scores the greater the QoL impairment.

We could observe this reduced QoL of the normosmic post-COVID patients even more clearly with the visual analogue scale. They had significantly higher scores than individuals in the control group. The hyposmic patients showed an even greater reduction in QoL.

There were no indications that the data could be unreliable, as the post-COVID patients even had a lower score in the QOD-DS questions than the control groups. The post-COVID patients thus continue to have a significant impairment of their QoL, even if the olfactory ability is back in the normosmic range. One reason for this could be the significantly younger age of the normosmic post-COVID patients compared to the healthy control group (*p* = 0.01), as olfactory ability decreases with age [15]. It could be assumed that this subgroup of post-COVID patients were already back in the official normosmic range, but not yet at baseline and thus felt more impaired. An additional modulating factor could be parosmia which was observed in both normosmic and hyposmic post-COVID patients. At least 10% of COVID patients complain of parosmia [3, 16] and an effect of parosmia on subjective QoL is well described in the literature [8, 10, 17]. Also in this study, the scores in the QOD-P and in QOD-QOL and QOD-VAS correlated.

However, it is particularly interesting to take a close look at the subpopulation of post-COVID patients who still had impaired olfactory function at the time of the study. Their QoL was decreased compared to the healthy control patients. This could be detected both by the QOD-QOL and the QOD-VAS, and it is not surprising considering the poorer olfactory function. However, it was shown that the hyposmic post-COVID patients did not have a worse QoL than hyposmic patients after other viral diseases and similar symptom duration. By contrast, these patients even showed significantly worse QoL, both in the QOD-QOL and QOD-VAS measures. QoL was not influenced by parosmia more than in other postviral olfactory disorders. This indicates that the decrease in olfactory QoL in post COVID patients is not due to a specific effect of the disorder but that it is more the olfactory loss itself that affects QoL, in this case largely independent of its cause.

Currently, there is a debate whether COVID-19-associated olfactory dysfunction is a separate entity or whether it behaves like other postviral olfactory disorder. The high expression of the surface molecule ACE-2 on the sustentacular cells led some authors to suspect a different pathomechanism than the direct viral attack on the sensory cells assumed for other postviral olfactory disorders, such as the parainfluenza virus [18, 19]. Olfactory disorders in COVID-19 are furthermore often not associated with symptoms of rhinitis and in this respect differ phenomenologically and probably also pathophysiologically from other viral olfactory disorders [20].

Following meta-analytical comparisons, other authors highlight the commonality of COVID-associated olfactory dysfunction with postviral olfactory dysfunction [21, 22]. The above-mentioned affection of sustentacular cells is also known from other viruses, such as influenza viruses, at least in animal models [19]. With regard to QoL, our observations show that it is limited in both entities. Whether it is actually less impaired in COVID patients than in other postviral olfactory disorders, as our data suggest, should be verified by further studies with a larger number of participants.

A clear limitation of this study is the diversity of the groups, which is due to the different recruitment of the groups − from the consultation for COVID-19 patients on the one hand and from an already existing database on the other hand. This led to significant differences in the age of the patients and in the group size. Furthermore, the post-COVID patients' group and the postviral controls included significantly more women than men. It is known that gender has an influence on QOD-P [8] and that women score higher than men when assessing olfactory dysfunction [23, 24] probably because they typically outperform men in terms of olfactory sensitivity [10].

The assessment of QoL in relation to other COVID-19-related complaints, e.g., a permanent reduction in lung capacity with dyspnoea, headaches and fatigue, memory, and sleep disorders [25, 26, 27, 28] should also be included in further studies. These additional complaints might have affected QoL as determined with the QOD. Here, other QoL questionnaires could be used. Also, longitudinal studies should be done, as this study is only a snapshot 6 months after infection. Still the present work adds to the small body of literature on COVID-19-associated olfactory dysfunction and its impact on QoL [29, 30].

## Statement of Ethics

Subjects in the study gave written informed consent to participate. The conduct of the study was approved by the Ethics Committee of the University Hospital of Cologne (20-1520).

## Conflict of Interest Statement

All the authors declare no competing interests.

## Funding Sources

There is no funding for this study.

## Author Contributions

Dr. M.S. Otte and Prof. J.C. Luers initiated the study. Dr. M.S. Otte and Ms. M.-L. Bork recruited and examined the patients. Prof. J.P. Klussmann, as clinic director, provided the financial resources. Prof. T. Hummel wrote and revised the paper with Dr. M.S. Otte. Prof. T. Hummel and Prof. A. Hähner provided the data of the control groups from a previously published dataset.

## Data Availability Statement

The data of the study are with the corresponding author. The subjects have consented to anonymized processing of the data and publication, but renewed consent must be requested for data disclosure.

## Figures and Tables

**Table 1:** Results of the olfactory test (TDI) and the assessment of the QoL with the QOD in post-COVID patients, healthy controls, and control patients with olfactory disorders of other viral etiology

	TDI	QOD-QOL	QOD-P	QOD-DS	QOD-VAS
Healthy controls					
Mean	33.80	7.94	1.28	11.69	5.78
*N*	32	32	32	32	32
SDM	±0.53	±1.15	±0.33	±0.55	±0.16
Postviral controls					
Mean	20.04	27.43	5.07	11.25	20.75
*N*	14	14	14	12	14
SDM	±1.59	±2.79	±0.75	±1.04	±4.57
Post-COVID patients					
Total					
Mean	32.90	8.91	2.50	8.48	11.34
*N*	65	44	44	44	44
SDM	±0.86	±1.37	±0.41	±0.33	±1.60
Normosmia					
Mean	35.37	7.69	2.36	8.47	9.25
*N*	53	36	36	36	36
SDM	±0.35	±1.40	±0.46	±0.39	±1.49
Hyposmia					
Mean	21.97	14.38	3.13	8.50	20.75
*N*	12	8	8	8	8
SDM	±2.77	±3.70	±0.93	±0.46	±4.57

The QOD consists of various sub-scores: QOD-QOL, QOD-P, QOD-DS, and QOD-VAS.

## References

[1] Haehner A, Draf J, Dräger S, de With K, Hummel T (2020). Predictive value of sudden olfactory loss in the diagnosis of COVID-19. ORL J Otorhinolaryngol Relat Spec.

[2] Luers JC, Rokohl AC, Loreck N, Wawer Matos PA, Augustin M, Dewald F (2020). Olfactory and gustatory dysfunction in coronavirus disease 19 (COVID-19). Clin Infect Dis.

[3] Parma V, Ohla K, Veldhuizen MG, Niv MY, Kelly CE, Bakke AJ (2020). More than smell − COVID-19 is associated with severe impairment of smell, taste, and chemesthesis. Chem Senses.

[4] Lechien JR, Chiesa-Estomba CM, Place S, Van Laethem Y, Cabaraux P, Mat Q (2020). Clinical and epidemiological characteristics of 1,420 European patients with mild-to-moderate coronavirus disease 2019. J Intern Med.

[5] Otte MS, Klussmann JP, Luers JC (2020). Persisting olfactory dysfunction in patients after recovering from COVID-19. J Infect.

[6] Otte MS, Bork ML, Zimmermann PH, Peter Klussmann JP, Luers JC (2021). Persisting olfactory dysfunction improves in patients six months after COVID-19 disease. Acta Otolaryngol.

[7] Hopkins C, Burges Watson DL, Kelly C, Deary V, Smith BC (2020). Managing long covid: don't overlook olfactory dysfunction. BMJ.

[8] Zou LQ, Hummel T, Otte MS, Bitter T, Besser G, Mueller CA (2021). Association between olfactory function and quality of life in patients with olfactory disorders: a multicenter study in over 760 participants. Rhinology.

[9] Oleszkiewicz A, Schriever VA, Croy I, Hähner A, Hummel T (2019). Updated sniffin' sticks normative data based on an extended sample of 9,139 subjects. Eur Arch Otorhinolaryngol.

[10] Frasnelli J, Hummel T (2005). Olfactory dysfunction and daily life. Eur Arch Otorhinolaryngol.

[11] Karamali K, Elliott M, Hopkins C (2022). COVID-19 related olfactory dysfunction. Curr Opin Otolaryngol Head Neck Surg.

[12] Boscolo-Rizzo P, Hummel T, Hopkins C, Dibattista M, Menini A, Spinato G (2021). High prevalence of long-term olfactory, gustatory, and chemesthesis dysfunction in post-COVID-19 patients: a matched case-control study with one-year follow-up using a comprehensive psychophysical evaluation. Rhinology.

[13] Croy I, Nordin S, Hummel T (2014). Olfactory disorders and quality of life: an updated review. Chem Senses.

[14] Jung YG, Lee JS, Park GC (2014). Does post-infectious olfactory loss affect mood more severely than chronic sinusitis with olfactory loss?. Laryngoscope.

[15] Cowart BJ (1989). Relationships between taste and smell across the adult life span. Ann N Y Acad Sci.

[16] Raad N, Ghorbani J, Safavi Naeini A, Tajik N, Karimi-Galougahi M (2021). Parosmia in patients with COVID-19 and olfactory dysfunction. Int Forum Allergy Rhinol.

[17] Bonfils P, Avan P, Faulcon P, Malinvaud D (2005). Distorted odorant perception: analysis of a series of 56 patients with parosmia. Arch Otolaryngol Head Neck Surg.

[18] Fodoulian L, Tuberosa J, Rossier D, Boillat M, Kan C, Pauli V (2020). SARS-CoV-2 receptors and entry genes are expressed in the human olfactory neuroepithelium and brain. iScience.

[19] Lee JC, Nallani R, Cass L, Bhalla V, Chiu AG, Villwock JA (2021). A systematic review of the neuropathologic findings of post-viral olfactory dysfunction: implications and novel insight for the COVID-19 pandemic. Am J Rhinol Allergy.

[20] Isenmann S, Haehner A, Hummel T (2021). Störungen der chemosensorik bei COVID-19: pathomechanismen und klinische relevanz. Fortschr Neurol Psychiatr.

[21] Imam SA, Lao WP, Reddy P, Nguyen SA, Schlosser RJ (2020). Is SARS-CoV-2 (COVID-19) postviral olfactory dysfunction (PVOD) different from other PVOD?. World J Otorhinolaryngol Head Neck Surg.

[22] Dicpinigaitis PV (2021). Post-viral anosmia (loss of sensation of smell) did not begin with COVID-19!. Lung.

[23] Cain WS (1982). Odor identification by males and females: predictions vs. performance 1. Chem Senses.

[24] Sorokowski P, Karwowski M, Misiak M, Marczak MK, Dziekan M, Hummel T (2019). Sex differences in human olfaction: a meta-analysis. Front Psychol.

[25] Garrigues E, Janvier P, Kherabi Y, Le Bot A, Hamon A, Gouze H (2020). Post-discharge persistent symptoms and health-related quality of life after hospitalization for COVID-19. J Infect.

[26] Jacobs LG, Gourna Paleoudis E, Lesky-Di Bari D, Nyirenda T, Friedman T, Gupta A (2020). Persistence of symptoms and quality of life at 35 days after hospitalization for COVID-19 infection. PLoS One.

[27] Taboada M, Moreno E, Cariñena A, Rey T, Pita-Romero R, Leal S (2021). Quality of life, functional status, and persistent symptoms after intensive care of COVID-19 patients. Br J Anaesth.

[28] van der Sar-van der Brugge S, Talman S, Boonman-de Winter L, de Mol M, Hoefman E, van Etten RW (2021). Pulmonary function and health-related quality of life after COVID-19 pneumonia. Respir Med.

[29] Coelho DH, Reiter ER, Budd SG, Shin Y, Kons ZA, Costanzo RM (2021). Quality of life and safety impact of COVID-19 associated smell and taste disturbances. Am J Otolaryngol.

[30] Elkholi SMA, Abdelwahab MK, Abdelhafeez M (2021). Impact of the smell loss on the quality of life and adopted coping strategies in COVID-19 patients. Eur Arch Otorhinolaryngol.

